# Cause or consequence? Exploring authors' interpretations of correlations between fish body condition and parasite infection

**DOI:** 10.1111/jfb.70048

**Published:** 2025-04-08

**Authors:** Ryota Hasegawa, Robert Poulin

**Affiliations:** ^1^ Graduate School of Environmental Science Hokkaido University Sapporo Japan; ^2^ Department of Zoology University of Otago Dunedin New Zealand

**Keywords:** body condition index, fish health, fisheries, helminth, parasite, physiological condition

## Abstract

We reviewed 194 publications that reported relationships between fish body condition indices (BCIs) and parasite infections, and examined the authors' intention behind this cross‐sectional analysis, that is, whether authors interpreted the negative correlations as the negative effects of parasites or as fish with poor BCIs being more susceptible to infections. While 89% of studies only considered parasite infections as causes of poor BCI, studies acknowledging the opposite or bidirectional causal links were rare. We recommend considering both possibilities in any given fish host and parasite association.

Fish species harbour various types of parasites, many of which can negatively affect host health under some conditions (Barber et al., 2000). To evaluate the potential negative effects of parasites on host health, fish biologists have historically used body condition indices (BCIs) based on body mass and length relationships (Jones et al., 1999). For instance, Fulton's BCI, or *K*, which is calculated as body mass/body length^3^, is probably the most widely recognized BCI in fish biology and fisheries (Nash et al., 2006). Researchers generally examine the relationships between BCI and parasite infections, such as by comparing BCIs between infected and uninfected fish, or regressing BCIs against parasite infection measures such as parasite abundance (number of parasites per fish) and parasite mass. Most researchers expect negative relationships between these variables (i.e. poorer body condition in infected or more heavily infected fish). Although this approach is useful for inferring the negative effects of parasites due to its cost‐effectiveness and simplicity, one concern arises: this cross‐sectional correlative approach cannot identify the hidden causal links underlying any observed correlation. Specifically, negative correlations between BCIs and parasite infections do not necessarily mean negative effects of parasites; they can also reflect increased susceptibility to infections in fish with poor body condition at the onset of the infection. This opposite causal link is highly plausible because poor body condition or poor nutritional states can greatly compromise an animal's costly anti‐parasitic defences, such as immune responses (Lochmiller, 1996). Indeed, several studies have identified that this causal link is highly prevalent in wild animal populations (Beldomenico et al., 2008; Blanchet, Méjean, et al., 2009; Godfrey et al., 2010; Hasegawa et al., 2025). Despite these facts, this opposite causal link has been largely ignored in empirical studies compared to the presumed negative effects of parasites on host body condition (Beldomenico & Begon, 2010). This is problematic because this biased interpretation could hinder our understanding of host–parasite relationships and lead to misleading conclusions, such as under‐ or overestimation of the negative effects of parasites.

In this study, we critically reviewed 194 publications examining the cross‐sectional correlations between fish BCIs and parasites infections. We specifically focused on how the authors of these studies have interpreted and reported these correlative methods and their results.

We used a subset of the dataset compiled by Hasegawa & Poulin (in press) (Data S1). In our previous study, we retrieved studies that potentially examined the relationships between fish BCIs and macroparasite infections from the ISI Web of Science on 26 October 2022 (see Hasegawa & Poulin in press). This earlier study focused on the biological drivers of the strength and direction of these relationships, whereas the present study focuses on how they are interpreted. In total, 809 publications were retrieved, and 216 of these publications were retained after a two‐step screening process (i.e. title and abstract screening, and full‐text screening; see Hasegawa & Poulin in press). All these publications either examined correlations between fish BCIs and measures of parasite infections, such as abundance, or compared fish BCIs among infection status, such as infected versus uninfected fish. Of these, we excluded the publications using an experimental approach, such as experimental infection or removal of parasites (*N* = 22), because these studies can more precisely identify causal relationships between BCIs and infections. Thus, the final dataset used here comprised 194 publications, all of which were conducted in field or aquaculture settings, such as fish farms.

We carefully scrutinized the main text of these studies and categorized them into four groups based on the authors' interpretations of fish BCI–parasite infection relationships: (I) studies that only considered parasites as a cause of poor body condition (i.e. parasites reduce body condition), with no mention of the alternative; (II) studies that only interpreted parasites as a consequence of poor body condition (i.e. fish in poor body condition are more likely to get infected); (III) studies that considered parasites as both a cause and a consequence of poor body condition; and (IV) studies that reported the correlations but did not discuss or interpret them, or interpreted these correlations as pseudo‐correlations (spurious relationships). Specifically, if authors used BCIs only to detect possible negative effects of parasites on host body condition, or if they only discussed negative effects of parasites based on their results (even if no significant negative relationship was found), we categorized such studies as (I). Although our previous study already revealed that most studies did not find significant negative correlations between BCIs and parasite infections (see Hasegawa & Poulin, in press), our preliminary assessment indicated that many researchers concluded that parasites had no effects on fish BCIs in these cases. These cases were also included in category (I). In contrast, if authors only discussed the possibility of high susceptibility to parasite infections in fish with poor body condition, we categorized such studies as (II). If authors mentioned and discussed the possibility that parasites may be either the cause or consequence of poor body condition, we categorized such studies as (III). Finally, if authors neither described the purposes of their analysis nor discussed their results, we categorized such studies as (IV). The category (IV) also includes publications where authors only considered correlations between BCIs and parasite infections as spurious (i.e. one environmental factor drives both BCIs and parasite infections resulting in an apparent but meaningless relationship between the latter two). To statistically test whether the distribution of studies among categories was non‐random, we performed a chi‐squared test, assuming an equal probability (i.e. 25%) for each of the four categories.

Of all 194 publications we examined, 87.6% (*N* = 170) and 11.9% (*N* = 23) of studies were conducted on fish collected in the wild and from aquaculture facilities (fish farms, etc.), respectively. Only one study (0.5%) examined fish BCIs and parasite infections in both field and aquaculture conditions. Among the 170 studies on fish collected in the wild, 61.8% (*N* = 105) were conducted in freshwater environments, while 38.2% (*N* = 65) were conducted in marine environments. Among the 23 studies on fish from aquaculture environments, 73.9% (*N* = 17) were conducted in freshwater environments, while 26.1% (*N* = 6) were conducted in marine environments.

As expected, 89.2% of these studies (*N* = 173) fell into category (I) where authors only considered that “parasites are a cause of poor body condition” as an explanation for the correlation (Figure 1). Only one study focused solely on the opposite causal link (category II), where authors considered that “parasites are a consequence of poor body condition” (0.5%, *N* = 1). Studies that mentioned the possibility that “parasites can be either causes and consequences of poor body condition” (category III) were relatively scarce (5.2%, *N* = 10). Studies that did not discuss the causal links or assumed the relationships were spurious correlations (category IV) were also rare (5.2%, *N* = 10). The distribution of studies among categories was significantly non‐random (chi‐squared test; *χ* ^2^ = 427.2, *df* = 3, *p* < 0.001).

**FIGURE 1:**
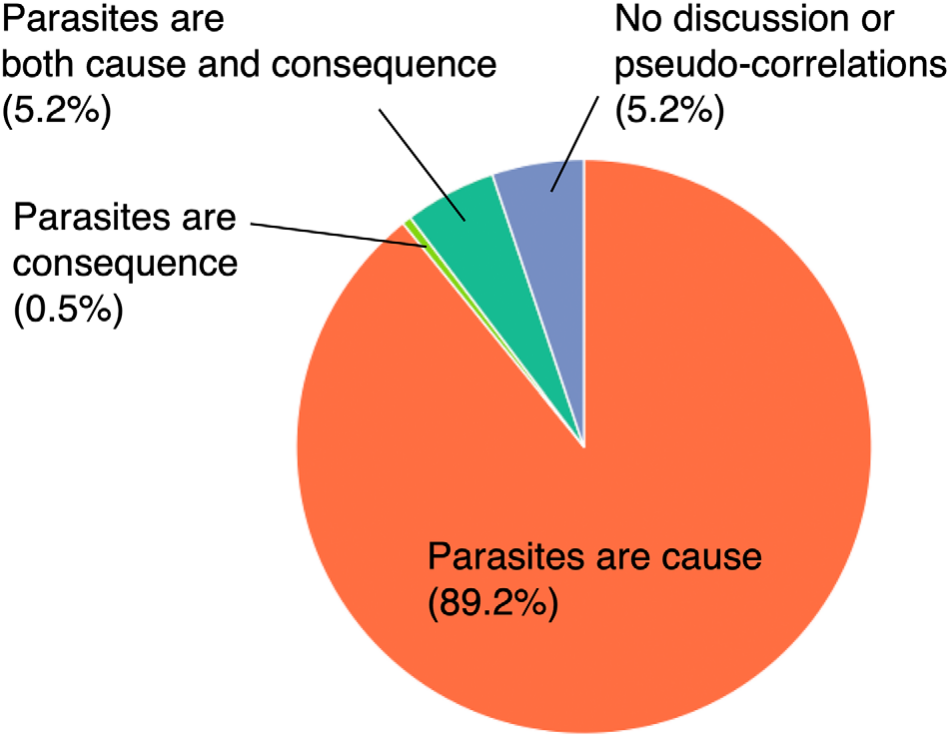
The proportion of studies in which the authors interpreted the relationships between fish body condition indices and parasite infections in each of four possible ways.

We provide the first evidence that many fish biologists have focused solely on the negative effects of parasites as an explanation for BCI–infection relationships, whereas the opposite causal link—high susceptibility to infections in fish with poor body condition—has been largely overlooked. This is highly problematic because these results suggest that many previous studies may have misinterpreted the mechanisms behind the association between parasite burden and BCIs. Specifically, even when parasites do not have detrimental effects and do not significantly reduce fish BCIs, negative relationships between BCIs and parasite infections can emerge because poor body condition may increase susceptibility to infections (Beldomenico et al., 2008; Blanchet, Thomas, & Loot, 2009). If researchers only consider parasites as causes of poor body condition in this context, they might overestimate how common the negative effects of parasites really are. Our data suggest this may be happening.

Most importantly, many researchers overlooked the possibility that parasites can be both causes and consequences of poor body condition (only 5.2% noted this). This is problematic because if both causal links occur simultaneously, a vicious circle or a positive feedback could arise: parasites might reduce host body condition, with poor body condition in turn leading to further parasite infections, and so on (Beldomenico et al., 2008; Beldomenico & Begon, 2010; Hasegawa et al., 2025). Such infection patterns could increase host mortality and have profound impacts on wild host populations (Beldomenico & Begon, 2010; Hasegawa et al., 2025). Indeed, some studies have provided evidence that both causal links are possible in wild populations (Beldomenico et al., 2008; Blanchet, Méjean, et al., 2009; Hasegawa et al., 2025). Vicious circles could be exacerbated in the context of environmental changes, such as climate change (Beldomenico & Begon, 2010, 2016; Hasegawa et al., 2025), because some environmental changes can reduce host body condition by inducing high stress, which can increase susceptibility to infections (Beldomenico & Begon, 2016). The negative effects of parasites on host health can also become stronger under unfavourable conditions for fish hosts (e.g. Thilakaratne et al., 2007). Therefore, it is essential to consider both causal links in any fish host and parasite systems, especially under the climate change scenarios predicted for the Anthropocene.

We should note that both causal links are unlikely to jointly occur in every host–parasite system; rather a single causal link is likely responsible for observed correlations in some, if not most, systems. For instance, both causal links are likely to occur in ectoparasite systems because infective larvae may easily attach to hosts with poor body condition due to their reduced mobility and poor immunity (Koprivnikar et al., 2012). In turn, the strong negative effects of such parasites on host body condition have been well‐documented in many ectoparasite systems (e.g. Forrester & Finley, 2022; Ooue et al., 2017). In contrast, in the case of trophically transmitted helminths, such as nematodes and cestodes, it may be difficult to detect high susceptibility to infections (i.e. parasites cannot be a consequence of poor body condition) because hosts acquire infections through feeding, which results simultaneously in better body condition and higher parasite abundance (e.g. Pascual et al., 2018). Due to this transmission mode, the negative effects of these helminths on BCIs are also generally unclear, except when host individuals are heavily infected (e.g. Blanchet, Méjean, et al., 2009). Nonetheless, exploring whether both causal links occur all kinds of host–parasite systems is crucial for identifying which systems suffer from the vicious circle described above.

In summary, we recommend the careful consideration of interpreting the relationships between BCIs and parasite infections, especially when authors discuss both causal links as possible explanations for any correlation between these two variables. Experimental approaches, such as experimental infections or parasite removal, could be the most rigorous methods to test causal links between fish BCIs and parasite infections (Blanchet, Thomas, & Loot, 2009). Another useful method could be longitudinal field monitoring of fish BCIs and parasite infections, for instance using capture‐mark‐recapture studies, which may allow inference of the causal underpinnings of any observed relationship (Beldomenico & Begon, 2010; Blanchet, Thomas, & Loot, 2009; Hasegawa et al., 2025). Moreover, it should be noted that not only both causal links but also spurious correlations (i.e. one factor drives both BCIs and parasite infections, resulting in an apparent but meaningless relationship between the latter two) may prevail in the correlations between fish BCIs and infections because both variables are influenced by numerous external factors (Sánchez et al., 2018). Considering the relationships between BCIs and parasite infections without bias is absolutely necessary to understand the true infection dynamics in host–parasite systems, which is itself essential for the management and conservation of fish stocks and the continued profitability of aquaculture.

## AUTHOR CONTRIBUTIONS

Conceptualization and writing, conducting the research and data interpretation: R.H. and R.P. Preparation of figures: R.H.

## FUNDING INFORMATION

This study was partially supported by a Japan Society for the Promotion of Science (JSPS) Research Fellow Grant (Grant No. JP22KJ0086 to RH) and a JSPS Overseas Research Fellow Grant (Grant No. 202460294 to RH).

## CONFLICT OF INTEREST STATEMENT

The authors declare no conflict of interests.

## Supporting information


**Data S1.** Supporting Information.

## Data Availability

The dataset supporting this article are available as Supporting Information.
